# ﻿*Persicariazhenaiguoi* (Polygonaceae, Persicarieae), an overlooked new species from Dabie Mountains, central China

**DOI:** 10.3897/phytokeys.253.144408

**Published:** 2025-02-25

**Authors:** Yi-Ming Wei, Zhen-Hua Zhu, Shu-Qing Lei, Bo Li

**Affiliations:** 1 College of Agronomy, Jiangxi Agricultural University, Nanchang 330045, China Jiangxi Agricultural University Nanchang China; 2 Center for Integrative Conservation, Xishuangbanna Tropical Botanical Garden, Chinese Academy of Sciences, Mengla 666303, China Xishuangbanna Tropical Botanical Garden, Chinese Academy of Sciences Mengla China

**Keywords:** Buckwheat family, morphology, ocrea, Polygonoideae, taxonomy

## Abstract

*Persicariazhenaiguoi* Bo Li, a remarkable new species discovered from the summit of Tiantangzhai Peak of Dabie Mountains in Hubei Province, central China, is described and illustrated. Based on its spicate inflorescences, ciliate ocreae, and the absence of prickles, the species is placed in P.sect.Persicaria. Within this section, *P.zhenaiguoi* most resembles *P.orientalis* and *P.viscosa* in gross morphology, particularly in the presence of densely spreading villus throughout. However, the new species can be easily distinguished from these two similar taxa by its nearly sessile leaves, which are lanceolate in shape with broadly cuneate to rounded bases, membranous ocrea with a small circle of green leaf-like wing, sparse and interrupted inflorescences, glabrous peduncles, greenish tepals, and ovoid achenes with grooved surfaces. Diagnostic characteristics, along with comparative photographs of the three related species, are provided, as well as a detailed morphological description and information on the distribution and habitat of the new species.

## ﻿Introduction

*Persicaria* Mill. is one of the largest genera in the buckwheat family (Polygonaceae) and belongs to the tribe Persicarieae, which also includes *Bistorta* Mill. and *Koenigia* L. (Galasso et al. 2009; Sanchez et al. 2011; Schuster et al. 2015). The genus includes approximately 100 species, primarily annual or perennial herbaceous plants, and is widely distributed around the world (Brandbyge 1993; Freeman 2005; Galasso et al. 2009). Within the genus, six sections have been proposed: sect. Persicaria, sect. Amphibia Tzvelev, sect. Cephalophilon (Meisn.) H.Gross, sect. Echinocaulon (Meisn.) H.Gross, and sect. Tovara (Adans.) H.Gross, and sect. Truelloides Tzvelev (Galasso et al. 2009). However, the sole species of sect. Truelloides Tzvelev, *P.bungeana* (Turcz.) Nakai ex Mori, has been tested to be a member of the sect. Persicaria in previous molecular phylogenetic analyses (Min et al. 2013; Zhai 2021) and the monophyly of the other five sections were always supported in molecular phylogenetic analyses (Kim and Donoghue 2008; Schuster et al. 2015; Cao et al. 2022, 2023).

Among these sections, species of sect. Persicaria can be distinguished from other *Persicaria* taxa by having a combination of characteristic features, including: usually glabrous stems without prickles, lanceolate simple leaves, tubular ocreae typically with truncate and ciliate apex, spicate inflorescences with few to many flowers, styles that are not deflexed and have a hooked apex, and *Persicaria*-type pollen grains with 20 circular pores and a reticulum (Hedberg 1946; Haraldson 1978; Brandbyge 1993; Li 1998; Li et al. 2003; Kim and Donoghue 2008). In China, 23 species were originally recorded in Polygonumsect.Persicaria (Mill.) Meisn. (≡ Persicariasect.Persicaria) (Li 1998; Li et al. 2003). However, following the exclusion of *P.amphibia* (L.) Gray (which belongs to sect. Amphibia), the inclusion of *P.bungeana* (which should be transferred from sect. Truelloides), and the addition of two recently described species, i.e., *P.wugongshanensis* Bo Li (Li 2014) and *P.lankeshanensis* T.J.Liang & Bo Li (Liang and Li 2014), there are now 25 species of sect. Persicaria recognized in China.

**Figure 1.**
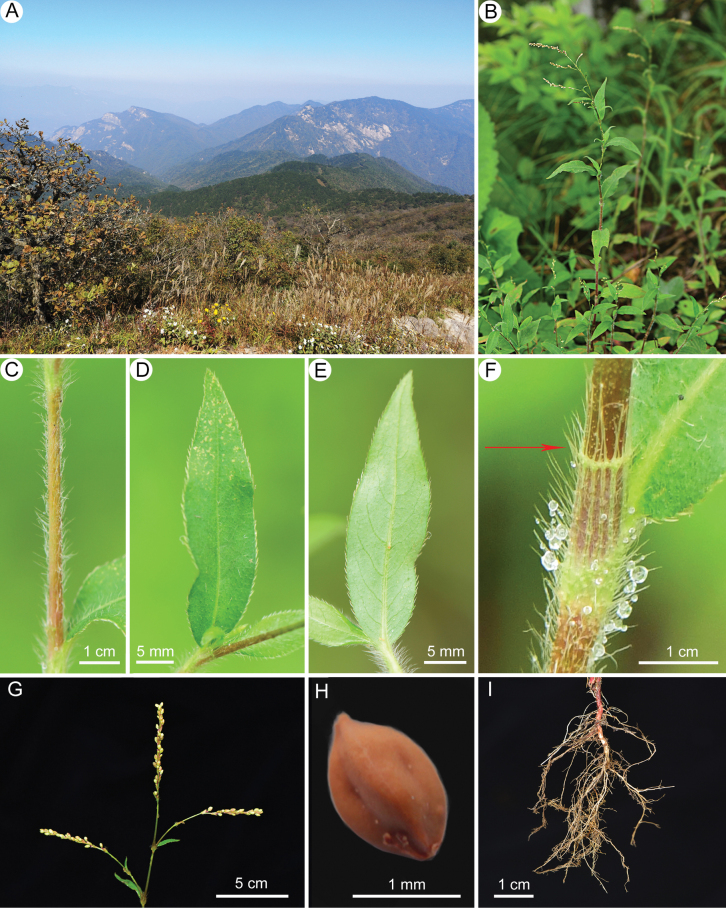
Habitat and morphology of *Persicariazhenaiguoi* Bo Li, sp. nov. **A** habitat **B** habit **C** stem **D** adaxial view of leaf blade **E** abaxial view of leaf blade **F** ocrea (the red arrow shows the green leaf-like wing) **G** inflorescence **H** achene **I** roots (photographed by Dr. Xin-Xin Zhu).

**Figure 2.**
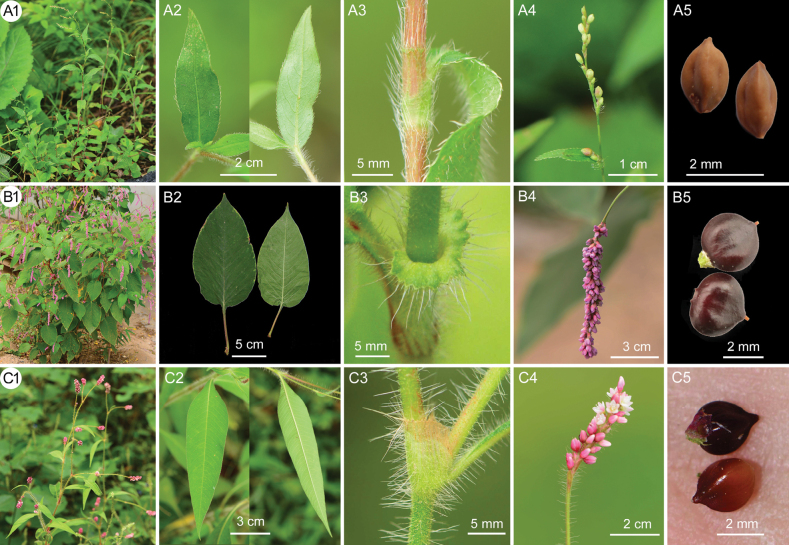
Morphological comparisons among *P.zhenaiguoi* (**A1–A5**), *P.orientalis* (**B1–B5**), and *P.viscosa* (**C1–C5**) **A1, B1, C1** habit **A2, B2, C2** leaves **A3, B3, C3** ocrea **A4, B4, C4** inflorescences **A5, B5, C5** achenes.

During field surveys in the Dabie Mountains of central China from 2022 to 2023, we encountered several populations of an unknown *Persicaria* plant at the summit of Tiantangzhai Peak (Fig. 1A) in Yingshan County of Hubei Province, which is morphologically remarkable in having dense spreading hairs throughout and a small ring of green, leaf-like wing at the apex of the ocrea (Fig. 1B–E). Based on its lanceolate leaves with broadly cuneate to rounded bases, spicate inflorescences, ciliate ocreae, and the absence of prickles, we confirmed that it is a member of the sect. Persicaria. After thorough morphological comparisons with congeneric taxa, a review of relevant literature, and examination of herbarium specimens, we found that the plant is most similar to *P.orientalis* (L.) Spach and *P.viscosa* (Buch.-Ham. ex D.Don) H.Gross ex Nakai but different in many aspects (Fig. 2). Thus, we have concluded that this plant represents a new, previously undescribed species of *Persicaria*, namely *P.zhenaiguoi* Bo Li, which is formally reported here.

## ﻿Materials and methods

Field surveys were carried out in the Dabie Mountains, located at the border between Hubei and Anhui provinces, central China, from June to September in 2022 and 2023, respectively. Morphological observations and descriptions of the putative new species were based on living plants in Yingshan County and herbarium specimens collected from the type locality. Its morphological variation was measured using a ruler and a micrometer. High-resolution images of type materials for all *Persicaria* taxa, including their synonyms, were consulted via JSTOR Plant Science (http://plants.jstor.org), and digital images of all *Persicaria* species recorded in China were examined through the Chinese Virtual Herbarium (https://www.cvh.ac.cn/). Morphological comparisons with *P.orientalis* and *P.viscosa* were critically evaluated using specimens deposited in CSH, GZTM, HBNU, HENU, HIB, IBK, IBSC, IMC, JJF, JMSM, JXCM, KUN, PE, QFNU, and SZ [acronyms according to (Thiers 2020)], as well as living plants that we have observed.

## ﻿Taxonomic treatment

### ﻿ Persicaria zhenaiguoi

Taxon classificationPlantaeCaryophyllalesPolygonaceae

Bo Li sp. nov.

4735CE23-4EAD-546A-B938-C135E09CFAC3

urn:lsid:ipni.org:names:77357225-1

Figs 1 , 2A1–A5

#### Diagnosis.

Morphologically, *P.zhenaiguoi* is superficially most similar to *P.orientalis* and *P.viscosa* in the indumentum characteristic, but can be clearly distinguished from the latter two taxa by its nearly sessile leaves, which are lanceolate in shape with broadly cuneate to rounded bases, membranous ocrea with a small circle of green leaf-like wing, sparse and interrupted inflorescences, glabrous peduncles, greenish tepals, and ovoid achenes with grooved surfaces (Fig. 2, Table 1).

**Table 1.** Comparison of morphological characteristics among *P.zhenaiguoi*, *P.orientalis* and *P.viscosa*.

	*P.zhenaiguoi*	*P.orientalis*	*P.viscosa*
Plant height	0.2–0.5 m	0.8–2.2 m	0.4–0.9 m
Stems	slender, erect, densely spreading villous	robust, erect, densely spreading villous	ascending to erect, densely spreading villous and glandular hairy, odoriferous
Petioles	connate with lower parts of ocrea, nearly absent	1.7–11.5 cm	3.3–1.2 cm, leaf base long decurrent along petioles
Leaf blades	lanceolate, 2.8–5.3 × 0.6–1.1 cm	broadly ovate, 10.5–22.8 × 5.1–12.3 cm	lanceolate, 4.4–7.8 × 1.2–2.1 cm
Ocreae	apex with a small circle of green leaf-like wing	apex usually with large green leaf-like wing	apex truncate
Inflorescences	erect, slender, interrupted below	pendulous, densely flowered	erect, densely flowered
Peduncles	glabrous	densely hirsute	densely spreading villous and glandular hairy
Perianth	greenish	pink or white	pinkish
Achenes	long ovoid, trigonous, surfaces grooved	nearly orbicular, biconcave, surfaces grooved	broadly ovoid, trigonous, surfaces flat

#### Type.

China • Hubei Province, Huanggang City, Yingshan County, Tiantangzhai Town, Dabie Mountains, in the grassland at the summit of Tiantangzhai Peak, 31°06'21.44"N, 115°46'22.60"E, alt. 1712 m, 10 July 2023, *X.X. Zhu et al. ZXX23818* (***holotype***: HITBC0122106, ***isotype***: IBSC0923376).

#### Description.

Herbs annual. Stems erect, slender, angulate, 20–50 cm tall, densely spreading villous. Petiole connate with lower parts of ocrea, nearly absent; Leaf blade lanceolate, 2.8–5.3 × 0.6–1.1 cm wide, apex acuminate, base broadly cuneate to rounded, margin densely ciliate, both surfaces densely villous, midvein slightly hollow on the adaxial surface and rise on the abaxial surface, lateral veins 8–10 pairs. Ocrea tubular, membranous, 0.6–1.1 mm long, densely villous, margin truncate with long ciliate and a small circle of green leaf-like wing. Inflorescence terminal or axillary, spicate, 2.8–5.1 cm long, slender, interrupted below, usually several spikes aggregated and panicle-like; peduncle glabrous. Bracts green, funnel-shaped, 2.8–4.6 mm long, sparsely villous, margin long ciliate, each 2–4 flowered. Pedicels slightly longer than bracts. Perianth greenish, 5-parted; tepals elliptic, 2.3–3.4 mm. Stamens 8, included. Styles 3, connate to below middle; stigma capitate. Achenes included in persistent perianth, 1.6–2.1 × 0.8–1.2 mm, brown, shiny, long ovoid, trigonous, surfaces grooved.

#### Phenology.

Flowering and fruiting was observed synchronously from July to October.

#### Etymology.

The specific epithet “*zhenaiguoi*” is dedicated to Professor Ai-Guo Zhen, in recognition of his significant contributions to the study of the local flora and biodiversity in Yingshan County.

#### Vernacular name.

The Chinese name of the new species is “甄氏蓼”, and the pronunciation of the Chinese Pinyin is zhēn shì liǎo.

#### Distribution and habitat.

**Figure 3.**
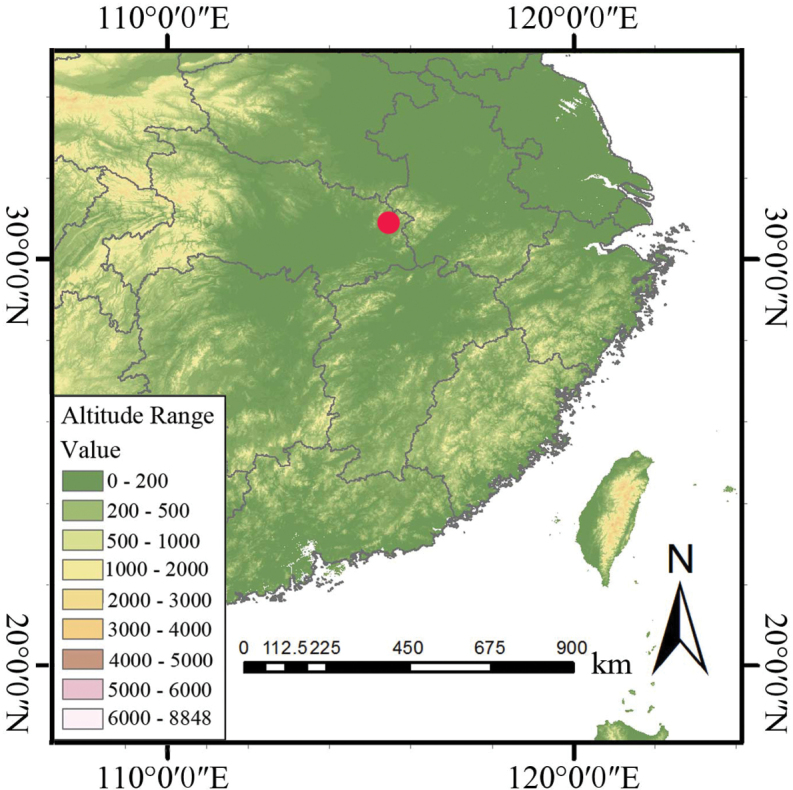
Distribution of *P.zhenaiguoi* (marked by the red circle).

*Persicariazhenaiguoi* can be found above an altitude of 1.600 m, in the moist grassland at the summit of Tiantangzhai Peak, one of the main peaks of the Dabie Mountains, which are located on the border between Hubei and Anhui provinces (Fig. 3). This plant is typically found growing alongside species of *Dryopteris* Adanson and *Carex* L.

#### Preliminary conservation status.

As currently known, this species has only been discovered from its type locality in the Tiantangzhai Peak of the Dabie Mountains, where it is distributed in a small area within the mountaintop region. Moreover, its habitat is increasingly threatened by the growing development of local tourism (author’s personal observation). Therefore, it should be categorised as critically endangered under criteria B and D following IUCN Red List Categories (IUCN 2012).

#### Taxonomic notes.

Morphologically, the ocrea of all species within P.sect.Persicaria is tubular and membranous, with a truncate, mostly ciliate apex (Meisner 1856; Gross 1913; Steward 1930; Haraldson 1978; Tutin et al. 1991; Li et al. 2003; Freeman 2005). Before this, *P.orientalis* was the only species in this section known to typically have a circle of green, leaf-like wing at the apex of the ocrea (Fig. 2B3). *P.zhenaiguoi* is the second species to exhibit a similar structure, caused by the enlargement of the longitudinal veins at the apex of the ocrea (Fig. 2A3), although not as prominent as observed in *P.orientalis*. They also share the similar indumentum that are long spreading white hairs densely covering on stems, leaves as well as ocreae, but are clearly different from each other in plant size (Fig. 2A1, B1), leaf shape and size (Fig. 2A2, B2), inflorescence structure (Fig. 2A4, B4), as well as the fruit shape (Fig. 2A5, B5). *Persicariaviscosa* is another species having the same indumentum and much more similar to *P.zhenaiguoi* in gross morphology, but it differs from the latter in several aspects: its leaf bases are long decurrent along petioles (Fig. 2C2), the peduncles cover dense hirsute and glandular hairs (Fig. 2C1, C4), and the inflorescences are dense and pinkish (Fig. 2C4). Detailed morphological comparisons among these three taxa are listed in the Table 1.

It is worthy to mention that the habitat and distribution of *P.zhenaiguoi* are somewhat unusual, at least when comparing with other species in sect. Persicaria found in central and southern China. Based on our years of observation, species from sect. Cephalophilon and sect. Echinocaulon, such as *P.nepalensis* (Meisn.) H.Gross, P.runcinatavar.sinensis (Hemsl.) Bo Li, and *P.thunbergii* (Siebold & Zucc.) H.Gross, are easily found in habitats like the summit of Tiantangzhai Peak (Fig. 1A). However, species from sect. Persicaria are rarely occurring at such high altitudes. At the same time, there has been a lack of comprehensive and in-depth surveys of the plant flora in the Dabie Mountains, especially in the mountaintop areas. This may explain why *P.zhenaiguoi* has likely been overlooked and not collected in the Dabie Mountains until now.

#### Additional specimens examined.

*Persicariaorientalis*: China • Anhui Province, Shucheng County, Xiaotian Town, 1 October 1951, *East China Workstation 4162* (PE00497378!); • Chongqing Municipality, Nanchuan County, Sanquan Village, alt. 640 m, 14 October 1985, *Z.Y. Liu 7429* (IMC0038461!); • Guangdong Province, Yangjiang City, Jiangcheng District, Hailing Town, 21°37'36.42"N, 111°58'8.87"E, alt. 27 m, 6 April 2021, *G.W. Tang et al. TangGW1343* (KUN1556765!); • Guizhou Province, Congjiang County, Doli Town, Panli Village, 25°35'14.35"N, 108°59'11.11"E, alt. 672 m, 21 September 2019, *K.T. Liu 522633190921914LY* (GZTM0096269!); • Hainan Province, Haikou City, Longhua District, Xue Village, 19°57'39.19"N, 110°20'57.89"E, alt. 15 m, 2 May 2018, *Y.T. Hou et al. 20180426580-1* (QFNU0048156!); • Hebei Province, Longhua County, Bugugou Town, alt. 993 m, 20 August 2013, *Group Six Z0272* (HBNU20001289!); • Heilongjiang Province, Qiqihar City, Longjiang County, 47°19'23"N, 123°11'41"E, alt. 131 m, 25 August 2019, *L.Y. Lin 2019082501* (JMSMC0000049!); • Henan Province, Xinxiang City, Huilong Village, 35°35'02"N, 113°35'57"E, alt. 369 m, 12 July 2018, *J.R. Li 368* (HENU1900368!); • Hunan Province, Nanyue District, Hengshan Nature Reserve, alt. 300 m, 1 September 2002, *Z.H. Hu 533* (PE00497445!); • Jiangsu Province, Suqian City, Siyang County, Longji Town, 33°20'40.36"N, 118°38'19.97"E, alt. 18 m, 3 August 2019, *Y.T. Hou et al. 20190803230-1* (QFNU0052420!); • Jiangxi Province, Fengchen City, Xiushi Town, grassland, 27°51'38.81"N, 115°53'15.04"E, alt. 423 m, 3 October 2019, *L. Cao 360981191003305LY* (JXCM0010118!); • Shandong Province, Zouping City, Xiyu Village, 36°47'17"N, 117°40'49"E, alt. 342 m, 4 August 2018, *J.L. Lan 201808037-1* (QFNU0047293!); • Shanxi Province, Wanrong County, Jiachun Town, 32°21'0"N, 110°37'0"E, alt. 566 m, 28 July 2020, *Y.J. Feng 201941325966* (QFNU0059553!); • Zhejiang Province, Jinyun County, Huzhen Town, Xiaxiang Village, 28°47'23"N, 120°12'58"E, alt. 194 m, 2 July 2013, *H.Y. Shou & Z.H. Wang SHY00909* (CSH0012891!).

*Persicariaviscosa*: China • Chongqing Municipality, Zhong County, Baishi Town, Huangjia Village, 30°20'17.78"N, 107°56'28.29"E, alt. 624 m, 30 May 2013, *Zhong County Team 500233-130530-519-03* (IMC0045792!); • Guangxi Province, Liuzhou City, Liujiang County, Jinde Town, Siliang Village, 24°15'44.59"N, 109°20'25.69"E, alt. 114 m, 14 August 2018, *Liujiang Team 450221180814022LY* (IBK00425346!); • Heilongjiang Province, Jiamusi City, Huachuan County, Shenjiadian Village, 46°34'39.68"N, 130°37'48.81"E, alt. 189 m, 8 September 2018, *C. Wang & Y.G. Peng WangCh532* (KUN1554918!); • Henan Province, Xinyang City, Shihe District, 32°6'52.15"N, 114°0'33.63"E, alt. 87 m, 8 June 2020, *X.X. Zhu et al. ZXX20942* (HIB0187735!); • Hubei Province, Hongan County, Xinhua Town, Zhangshan Village, alt. 80 m, 14 June 2019, *C.M. Tan et al. 19061418* (JJF00044259!); • Hunan Province, Shaoyang City, Dongkou County, alt. 350 m, 12 August 2004, *L.D. Duan 5337* (PE00640285!); • Jiangxi Province, Jiujiang County, alt. 250 m, 17 September 2005, *A.M. Dong 930* (SZG00002585!); • Jilin Province, Baishan City, Badaojiang District, 41°34'12.01"N, 126°34'33.24"E, alt. 311 m, 22 August 2019, *C.Q. Cao CaoChQ495* (KUN1487264!); • Yunnan Province, Baoshan City, Tengchong County, Qushi Town, 25°23'58"N, 98°50'54"E, 1820 m, 3 October 2009, *Y.F. Chen et al. 09209-3* (QFNU0056773!); • Shandong Province, Yantai City, Rizhao Village, 36°48'5"N, 121°18'55"E, alt. 18 m, 28 September 2015, *X.W. Xin Lilan859* (KUN1438272!).

## Supplementary Material

XML Treatment for Persicaria zhenaiguoi

## ﻿Additional information

### Author ORCIDs

Bo Li https://orcid.org/0000-0003-1628-8128

### Data availability

All of the data that support the findings of this study are available in the main text.

## References

[B1] Brandbyge J (1993) Polygonaceae. In: Kubitzki K Bittich V (Eds) The Families and Genera of Vascular Plants.Springer Verlag, Berlin, 531–544. 10.1007/978-3-662-02899-5_63

[B2] Cao DL Zhang XJ Qu XJ Fan SJ (2022) Plastid phylogenomics sheds light on divergence time and ecological adaptations of the tribe Persicarieae (Polygonaceae). Frontiers in Plant Science 13: 1046253. 10.3389/fpls.2022.1046253 PMC9780030 36570890

[B3] Cao DL Zhang XJ Qu XJ Fan SJ (2023) Phylogenomics, divergence time estimation, and adaptive evolution in the Polygonoideae (Polygonaceae). Journal of Systematics and Evolution 61(6): 1004–1019. 10.1111/jse.12946

[B4] Freeman CC (2005) Polygonaceae. In: Flora of North America Editorial Committee (Eds) Flora of North America.Oxford University Press, New York, 574–594.

[B5] Galasso G Banfi E Mattia FD Grassi F Sgorbati S Labra M (2009) Molecular phylogeny of *Polygonum* L. s.l. (Polygonoideae, Polygonaceae), focusing on European taxa: Preliminary results and systematic considerations based on rbcL plastidial sequence data. Atti della Società Italiana di Scienze Naturali e del Museo Civico di Storia Naturale di Milano 150(1): 113–148.

[B6] Gross H (1913) Beiträge zur Kenntnis der Polygonaceae. Botanische Jahrbücher für Systematik, Pflanzengeschichte und Pflanzengeographie 49: 234–339.

[B7] Haraldson K (1978) Anatomy and taxonomy in Polygonaceae subfam. Polygonoideae Meissn. [sic.] emend. Jaretzky. Symbolae Botanicae Upsalienses 22: 1–95.

[B8] Hedberg O (1946) Pollen morphology in the genus *Polygonum* L. s.l. and its taxonomic significance. Svensk Botanisk Tidskrift 40: 371–404.

[B9] IUCN (2012) IUCN Red List Categories and Criteria, Version 3.1 (2^nd^ edn.). Gland and Cambridge, 32 pp.

[B10] Kim ST Donoghue MJ (2008) Molecular phylogeny of *Persicaria* (Persicarieae, Polygonaceae). Systematic Botany 33(1): 77–86. 10.1600/036364408783887302

[B11] Li AJ (1998) Polygonaceae. In: Li AJ (Ed.) Flora Reipublicae Popularis Sinicae.Science Press, Beijing, 1–237.

[B12] Li B (2014) *Persicariawugongshanensis* (Polygonaceae: Persicarieae), an odoriferous and distylous new species from Jiangxi, eastern China. Phytotaxa 156(3): 133–144. 10.11646/phytotaxa.156.3.4

[B13] Li AJ Bao BJ Grabovskaya-Borodina AE Hong SP McNeill J Mosyakin SL Ohba H Park CW (2003) Polygonaceae. In: Li AJ (Ed.) Flora of China.Science Press, Beijing; Missouri Botanical Garden Press, St. Louis, 277–350.

[B14] Liang TJ Li B (2014) *Persicarialankeshanensis* (Polygonaceae: Persicarieae), a new species from Guangdong, China. Bangladesh Journal of Plant Taxonomy 21(2): 147–152. 10.3329/bjpt.v21i2.21353

[B15] Meisner CF (1856) Polygonaceae. In: de Candolle A (Ed.) Prodomus systematis naturalis regni vegetabilis.Masson Press, Paris, 1–185.

[B16] Min YJ Zhou ZZ Zhao XX Gao P Long C (2013) Phylogenetic position of *Polygonumbungeanum* in *Polygonum* L. s. lat. (Polygonaceae) as evidenced from nrDNA ITS, cpDNA *atpB-rbcL* and *trnL-F* sequences. Life Science Journal 10(2): 2664–2670.

[B17] Sanchez A Schuster TM Burke JM Kron KA (2011) Taxonomy of Polygonoideae (Polygonaceae): A new tribal classification. Taxon 60(1): 151–160. 10.1002/tax.601013

[B18] Schuster TM Reveal JL Bayly MJ Kron KA (2015) An updated molecular phylogeny of Polygonoideae (Polygonaceae): Relationships of *Oxygonum*, *Pteroxygonum*, and *Rumex*, and a new circumscription of *Koenigia*. Taxon 64(6): 1188–1208. 10.12705/646.5

[B19] Steward AN (1930) Polygoneae of Eastern Asia. Gray Herbarium of Harvard University Publishing, 1–132. http://www.jstor.org/stable/41764079

[B20] Thiers B (2020) [+Continuously updated]. Index herbariorum: a global directory of public herbaria and associated staff. New York Botanical Garden. http://sweetgum.nybg.org/science/ih/ [accessed 21 July 2024]

[B21] Tutin TG Burges NA Edmondson JR Heywood VH Moore DM Valentine DH Waiters SM Webb DA (1991) Polygonaceae. In: Tutin TG (Ed.) Flora Europaea (2nd edn.). Cambridge University Press, London, 91–108.

[B22] Zhai SS (2021) Study on taxonomy and phylogeny of *Persicaria* in China. Shandong Normal University Publishing, 1–128.

